# Extreme Procalcitonin Elevation without Proven Bacterial Infection Related to Amphetamine Abuse

**DOI:** 10.1155/2014/179313

**Published:** 2014-03-11

**Authors:** András Lovas, Zsuzsanna Ágoston, Klára Késmárky, Péter Hankovszky, Zsolt Molnár

**Affiliations:** ^1^Department of Anaesthesiology and Intensive Therapy, University of Szeged, Semmelweis Utca 6, Szeged 6722, Hungary; ^2^Département d'Anesthésiologie, Hôpitaux Universitaires de Genève, 4 rue Gabrielle Perret-Gentil, 1211 Geneva 14, Switzerland

## Abstract

Systemic inflammatory response with rhabdomyolysis and consequent multiorgan failure is a known sequela of psychotropic drug abuse. However, in cases with uncertain past medical history the initial diagnosis can be challenging. Here we report the case of a 21-year-old male who was admitted to the intensive care unit with severe neurological impairment caused by amphetamine intoxication. First laboratory investigations revealed extremely high serum procalcitonin (PCT) levels reaching a maximum concentration of 1640 ng/mL on the second day of observation. Although PCT has high sensitivity and specificity in differentiating bacterial sepsis from nonbacterial inflammation, our case report shows for the first time that it can be extremely elevated following serious amphetamine intoxication without bacterial infection.

## 1. Introduction

Procalcitonin (PCT) is a well-known biomarker for the diagnosis of sepsis. Although its absolute value and its kinetics over time have high sensitivity and specificity for differentiating bacterial infection from systemic inflammation in the critically ill [1, 2] but it is also important to interpret the results of the first measurement in the context of the full medical picture. One has to take into account that one cut-off value for PCT cannot be applied in all circumstances. Indeed, “elevated” PCT levels were described in surgical and trauma patients without proven infection but this increment was generated by the systemic inflammatory response syndrome (SIRS) in answer to the injurious insult [3, 4]. Severe SIRS can also be present in various medical conditions including amphetamine overdose [5]. We report the case of an extreme elevation of PCT without bacterial sepsis in a young man who presented with altered level of consciousness due to amphetamine intoxication which was initially thought to be infectious meningitis.

## 2. Case Report

The 21-year-old male was found unconscious by his parents at home. The attending ambulance crew measured severe hypoglycaemia. After the serum glucose level was normalised by iv. glucose infusion the patient was transferred to the Accident and Emergency Department with oxygen supplementation via a face mask. Regarding his medical history he was fit and well, not on any regular medication.

On the first assessment he was tachypnoeic (30/min) and had a SpO_2_ of 98%, sinus tachycardia (135/min), and a blood pressure of 96/67 mmHg. His neurological examination showed a Glasgow Coma Scale of 9 with no meningeal signs, dilated, equal pupils reacting for light, bulbs fixed to the left, and right sided hemiparesis predominantly on the lower limb. Pressure sore of the skin was found on the right flank and over the sacral region. Axillary temperature was 39.6°C and his minimal amount of urine looked concentrated after catheterisation. Arterial blood gases showed severe metabolic acidosis and hyperkalaemia. Apart from sinus tachycardia 12-lead ECG was normal.

The urgent computer tomography scan of the head and the cervical spine could not show any pathology. Soon after the diagnostic procedures the patient became very agitated and eventually endotracheal intubation had to be performed with rapid sequence induction after which the patient was transferred to the intensive care unit (ICU). Routine intensive care with mechanical ventilation, continuous propofol and morphine sedation, and standard monitoring was commenced.

Immediately after ICU admission arterial and central venous catheters were inserted and fluid resuscitation was started with crystalloid infusion. Cerebrospinal fluid (CSF), bronchial secretions, urine samples, and blood cultures were taken for microbiological examination and combined imipenem (500 mg tds); amikacin (1000 mg qd) antibiotic therapy was initiated. Due to the oliguria, severe metabolic acidosis, and hyperkalaemia, a hemodialysis catheter was inserted and intermittent venous-venous hemodiafiltration (IVVHDF) was started.

Analysis of the CSF sample revealed no leukocytosis; total protein level and the CSF to serum glucose were also normal. The appearance of the spinal fluid was clear and the latex agglutinin rapid diagnostic tests for* N. meningitidis, S. pneumoniae, *and* H. influenzae *were all negative. According to toxicology, ethanol was not detected in the serum but amphetamine was present in the urine sample.

Routine laboratory tests revealed acute kidney injury (creatinine: 395 *μ*mol/L, blood urea nitrogen: 21.5 mmol/L) most likely due to rhabdomyolysis indicated by extremely high creatinine kinase (Table 1). The rest of the most relevant laboratory parameters is summarised in Table 1. Patient rendered an APACHE II score of 34 points.

Microbiology did not confirm infection from the CSF, blood and urine samples, and in the view of the full clinical picture, and laboratory profile antibiotics were stopped after 24 hours.

The patient's general condition stabilised and on day 3 he was extubated. He remained agitated, which was treated successfully by iv. haloperidol boluses. The right sided hemiparesis also improved gradually with physiotherapy in which symptom was supposed to be the consequence of the prolonged immobilisation on the ground before hospital admission. However, he remained anuric and required daily IVVHDF for the rest of his stay in ICU. On day ten he was discharged with stable vital parameters and no focal neurological signs.

## 3. Discussion

Differentiating SIRS from bacterial sepsis remains a challenging task in the critically ill. Biomarkers like C-reactive protein, interleukin-6, and PCT can help the differential diagnosis but their sensitivity and specificity are far from 100 percent [6]. Furthermore, in cases of emergencies the medical history is often missing or lacking; therefore physicians can only rely on detailed physical assessment, radiology, and laboratory results. However, psychotropic drugs like amphetamine can cause tachycardia, hyperpyrexia, even multiorgan failure, which may mimic severe sepsis and septic shock [7]. Furthermore, amphetamine consumption is well below the international figures in this region of Hungary; therefore clinicians do not have the routine in suspecting overdose in these cases [8].

Hyperpyrexia and concomitant rhabdomyolysis are related to impaired thermoregulation by dopaminergic and serotoninergic pathways and a direct toxic effect on muscle tissue [7, 9]. Liver dysfunction has also been described as part of hyperpyrexia induced by SIRS [7]. Rapid cooling with ongoing organ support has a paramount importance in the initial period of resuscitation and the cessation of further impairment.

Although PCT has been investigated extensively in bacterial infections it may also be elevated when bacterial infection is absent. Pancreatitis, burn injury, mechanical trauma, extended surgery, or even heatstroke are all well-known conditions where PCT levels may be increased [10]. In mechanical trauma where rhabdomyolysis may occur the serum concentration of PCT rapidly increases within 2 to 4 hours and peaks within 2 days but seldom outstrips the 10–20 ng/mL range even in the most severe cases [10]. Our case with the maximum serum concentration of 1640 ng/mL highlights the importance of careful interpretation of one single measurement regardless how high it may be. However, the kinetics of PCT followed the well-described pattern of daily decrease by roughly 50% as determined by its half-life (Table 1).

It is difficult to fully explain the extremely high PCT values but there are several factors, which may play a role in the process. It has been reported that PCT is produced on one hand from organs due to tissue injury and on the other hand from immune cells [11]. In the current case the tissue component had to play the major role in the release as we could not prove any infection, although the high leukocyte count may also indicate increased PCT release from macrophages. However, after surgery and trauma the increase in PCT is several folds less than what we observed in this patient. One of the possible explanations is that tissue injury was substantially more severe in our case than even after trauma, causing severe rhabdomyolysis and acute renal failure as a consequence.

However, our patient developed severe AKI with anuria requiring IVVHDF therapy; the renal excretion of PCT plays a minor role in the elimination and accumulation has not been observed in patients with renal impairment [12]. Level et al. also investigated the PCT elimination by hemofiltration but no significant serum level modification was described [12]. The slightly elevated troponin-T level found in this patient without proven myocardial ischemia can be explained again with the enormous skeletal muscle injury and partly by the acute renal failure [13].

PCT has also been used for guiding the length of antibiotic therapy [2]. In our case, despite the very high PCT levels, taking the full clinical picture into account and the lack of evidence of infection made us to stop antibiotic therapy on day 2.

## 4. Conclusion 

The extreme elevation of PCT without concomitant bacterial sepsis made this case particularly interesting. Such an extreme PCT elevation related to amphetamine intoxication has never been described previously. This case showed that even extremely high PCT levels may not indicate infection in patients with amphetamine overdose which caused rhabdomyolysis and acute kidney injury.

## Figures and Tables

**Table 1:** Blood chemistry results and their kinetics during stay in intensive care unit.

	Reference range	Day 1	Day 2	Day 4	Day 6	Day 10
PCT (ng/mL)	<0.5	1432	1640	1007	170.6	15.18
CRP (mg/L)	<5	8.5	n/a	n/a	n/a	n/a
WCC (cells/*μ*L)	3700–9500	22690	18160	13960	11250	15950
PLT (cells/*μ*L)	143000–332000	340000	204000	120000	115000	405000
INR		1.33	1.61	1.22	n.a.	n/a
LDH (U/L)	<530	2010	4754	7240	5150	n/a
GOT (U/L)	<37	651	2016	4207	1442	n/a
GPT (U/L)	<40	144	384	2016	463	n/a
CK (U/L)	<195	42960	125500	92700	20580	1325
Trop-T (*μ*g/mL)	<0.04	0.117	0.192	n/a	n/a	n/a

PCT: procalcitonin; CRP: C-reactive protein; WCC: white cell count; PLT: platelet count; INR: international normalised ratio; LDH: lactate dehydrogenase; GOT: glutamic oxaloacetic transaminase; GPT: glutamic pyruvic transaminase; CK: creatine kinase; Trop-T: troponin-T.
